# Blocking Retinal Chloride Co-Transporters KCC2 and NKCC: Impact on Direction Selective ON and OFF Responses in the Rat’s Nucleus of the Optic Tract

**DOI:** 10.1371/journal.pone.0044724

**Published:** 2012-09-06

**Authors:** Katharina Spoida, Claudia Distler, Anne-Kathrin Trampe, Klaus-Peter Hoffmann

**Affiliations:** 1 Allgemeine Zoologie and Neurobiologie, Ruhr-Universität Bochum, Bochum, Germany; 2 Tierphysiologie, Ruhr-Universität Bochum, Bochum, Germany; University College London, United Kingdom

## Abstract

In the present study we investigated *in vivo* the effects of pharmacological manipulation of retinal processing on the response properties of direction selective retinal slip cells in the nucleus of the optic tract and dorsal terminal nucleus (NOT-DTN), the key visuomotor interface in the pathway underlying the optokinetic reflex. Employing a moving visual stimulus consisting of either a large dark or light edge we could differentiate direction selective ON and OFF responses in retinal slip cells. To disclose the origin of the retinal slip cells' unexpected OFF response we selectively blocked the retinal ON channels and inactivated the visual cortex by cooling. Cortical cooling had no effect on the direction selectivity of the ON or the OFF response in NOT-DTN retinal slip cells. Blockade of the retinal ON channel with APB led to a loss of the ON and, to a lesser degree, of the OFF response and a reduction in direction selectivity. Subsequent blocking of GABA receptors in the retina with picrotoxin unmasked a vigorous albeit direction unselective OFF response in the NOT-DTN. Disturbing the retinal chloride homeostasis by intraocular injections of bumetanide or furosemide led to a loss of direction selectivity in both the NOT-DTN's ON and the OFF response due to a reduced response in the neuron's preferred direction under bumetanide as well as under furosemide and a slightly increased response in the null direction under bumetanide. Our results indicate that the direction specificity of retinal slip cells in the NOT-DTN of the rat strongly depends on direction selective retinal input which depends on intraretinal chloride homeostasis. On top of the well established input from ON center direction selective ganglion cells we could demonstrate an equally effective input from the retinal OFF system to the NOT-DTN.

## Introduction

In all mammals investigated so far a common pathway underlying the horizontal optokinetic reflex (hOKR) has emerged. Retinal slip neurons in the pretectal nucleus of the optic tract and the dorsal terminal nucleus of the accessory optic system (NOT-DTN) represent the visuomotor interface linking the visual input from the retina and, in many mammals e.g. rat [1], rabbit [2], ferret [3], cat [4], guinea pig [5], monkey [6], but not in marsupials [7], [8], the visual cortex with the motor output innervating the extraocular muscles via relays in the brainstem, the cerebellum, and the deep cerebellar nuclei [9, for a recent review see 10]. Characteristically, retinal slip neurons code for the discrepancy between velocity of the stimulus movement and eye velocity, i.e. the retinal slip. They are strongly selective for ipsiversive stimulus movement, i.e. neurons in the left NOT-DTN prefer movement to the left, and *vice versa*. On the basis of the broad velocity range NOT-DTN cells respond to it is thought that they are driven by retinal input from both ON- and ON-OFF direction selective ganglion cells [9], [11], [12]. Anatomical, electrophysiological, and pharmacological studies have suggested that the other nuclei in the accessory optic system receive input only from ON-center direction selective ganglion cells in all vertebrates investigated so far (turtle [13, 14, 15, mouse [16], rat [17], rabbit [18], [19], cat [20]). In addition, specific subtypes of ON/OFF direction selective ganglion cells have been recently identified to differentially project to the NOT and the medial terminal nucleus (MTN) in mice [21]. Despite numerous studies during the last decade the exact mechanisms creating direction selectivity in the retina and in the NOT-DTN remain elusive. Nevertheless, it seems quite clear now that starburst amacrine cells that simultaneously release GABA and acetylcholine as neurotransmitters are critically involved in retinal direction selectivity. Early experiments on the systemic level have shown that eliminating starburst amacrine cells pharmacologically leads to a loss of direction selectivity in the nucleus of the basal optic root and to a loss of OKR in chicken [22], [23], mouse [24], and rabbit [25]. Starburst amacrine cells are characterized by radially symmetric dendritic trees that convey directionally selective GABAergic inhibition to direction selective ganglion cells [26], [27], [28]. Indeed, these dendrites themselves are already direction selectively depolarised by movements from the soma to the tip of the dendrite. Evidently, this dendritic direction selectivity is based on voltage gated channels, dendritic voltage gradients, and an asymmetric chloride equilibrium potential created by asymmetric distributions of cation-chloride-cotransporters [28], [29], [30], [31], [32]. Direction selectivity in ganglion cells is then generated by spatially asymmetric input-output relationships of amacrine dendrites, lateral inhibition between neighbouring amacrine cells, and local postsynaptic signal processing [28], [30], [31], [33], [34], [35], [36].

In the present study we sought to manipulate the retinal input to the NOT-DTN by intraocular injections of specific blockers of cation-chloride cotransporters to test which consequences of the loss of the dendritic direction selectivity in starburst amacrine cells achieved by this treatment [30] manifest themselves in the NOT-DTN responses and thus in the optokinetic system. New reports indicate that not only ON but also ON/OFF ganglion cells project to the NOT-DTN [21]. In addition, an OFF response can be unmasked in retinal direction selective ganglion cells by blocking GABA [37]. Therefore, we employed a visual stimulus containing moving dark or light edges not usually applied to the optokinetic system in order to differentiate ON and OFF responses in retinal slip cells, and to evaluate the effects of pharmacological manipulation on these responses separately.

## Methods

### Animals

All experiments were approved by the local authorities (Regierungspräsidium Arnsberg) and were performed according to the Deutsche Tierschutzgesetz of 7.26.2002, the European Communities Council Directive RL 2010/63/EC, and the NIH guidelines for care and use of animals for experimental procedures. Data were collected from 24 adult Long Evans rats of both sexes ranging in weight from 250 g to 490 g. All animals were bred and raised in the animal facility of the Department of Zoology and Neurobiology in an enriched environment.

### Surgery

After premedication with 0.05mg atropine sulphate (Braun) the animals were initially anaesthetized with ketamine hydrochloride (90 mg/kg, Ketamin® 10%, Pharmanovo) and xylacine hydrochloride (5 mg/kg, Rompun® 2%, Bayer). Deep analgesia was ensured by the intramuscular application of an initial bolus of 3 µg/kg fentanyl citrate (Fentanyl ®, Janssen-Cilag) and by a subcutaneous infusion of 3 µg/kg/h during the entire experiment. After additional local anaesthesia with bupivacain hydrochloride (Bupivavain® 0.5%, Jenapharm) a tracheotomy was carried out. The skin overlying the skull was cut and a craniotomy was performed to allow access to the visual cortex, the superior colliculus and the pretectum. In addition, a head holder was fixed on the skull to free the head from the ear bars and allow unobstructed vision during the experiment. At the end of surgical procedures, the animals received urethane (1 g/kg) intraperitoneally and were paralyzed with alcuronium chloride (Alloferin®, Valeant Pharmaceuticals). During the experiment, the animals were artificially ventilated with air, the endtidal CO_2_, heart rate, and body temperature were monitored and kept at physiological levels. Pupils were dilated with atropine, corneae were protected with a thin film of silicon oil. At the end of the experiment, the animals were sacrificed with an overdose of pentobarbital.

### Visual stimulation

The retinal slip cells in the NOT-DTN were localized, first, by their location lateral to the anterior border of the SC, i.e. the representation of the ipsilateral visual field in the SC, and second, by their characteristic preference for ipsiversive stimulus motion.

**Figure 1:**
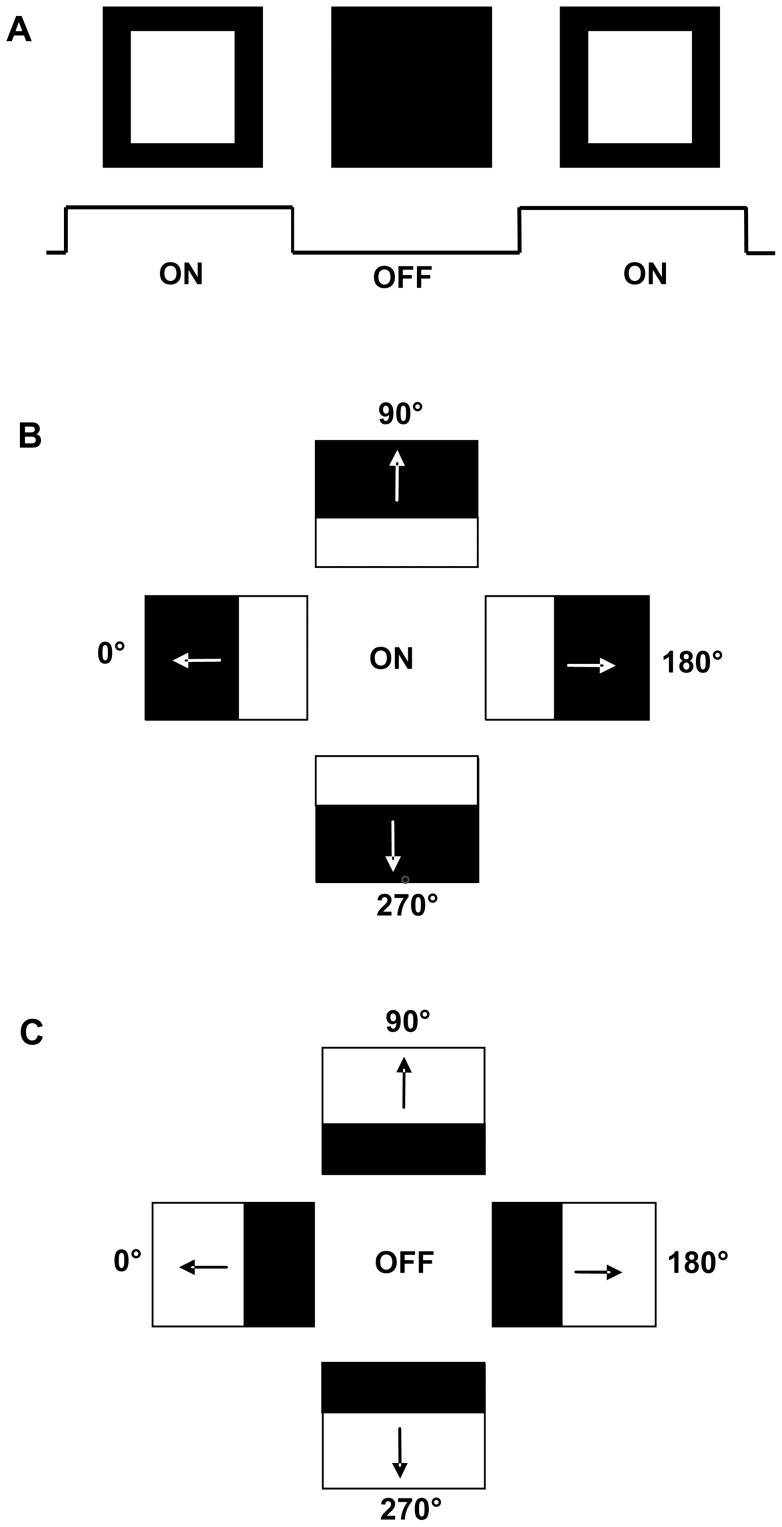
Schematic demonstration of the visual stimuli used in the present study. A: stationary flash stimulus, duration of the presentation of the bright (ON) and the dark (OFF) monitor lasted for 1s each. B: moving ON stimulus. A light edge moved across the dark monitor until it was fully bright in the four cardinal directions. C: moving OFF stimulus. A dark edge moved across the bright monitor until it was fully dark in the four cardinal directions (for further description see methods).

Visual stimuli were created with Cortex and Matlab® (version 7.2.0, The Math Works) and presented on a computer monitor (Dell, Model 2001FP, 17in, 640×480 Px, 60Hz) 25 cm in front of the animal. Various stimuli were used. A stationary flash stimulus consisting of a white square (120 cd/m^2^) on dark background (ON) and a dark background (<1 Cd/m^2^) (OFF) each lasting for 1s was used to characterize the unit's ON/OFF response (Fig. 1A). In addition, the neurons' response to moving stimuli was investigated with light (ON) or dark (OFF) edges (contrast 0.9) moving across a dark (or light background, respectively), in both vertical and horizontal directions at stimulus velocities between 5°/s and 40°/s. To be more explicit, in case of e.g. a light edge (ON stimulus) moving down, the screen was dark in the beginning, the light edge started at the top and moved down like a “light curtain” until the whole screen was bright (Fig. 1B). In case of a dark edge (OFF stimulus) moving down, the screen was bright in the beginning, the dark edge started at the top and moved down like a “dark curtain” until the whole screen was dark (Fig. 1C). In some cases, a sinusoidal grating (spatial frequency 0.05°, repetition rate 1°/s), or a random dot pattern moving in eight directions was used.

### Intraocular injections

After a retinal slip cell had been localized measurements were carried out to characterize the unit's ON/OFF response and directional preference. Under additional anaesthesia with 1.5–2% halothane, one of various pharmacological substances was then injected into the eye contralateral to the recorded NOT-DTN. The retinal ON-response was blocked with 2-amino-4-phosphonobutyrate (APB, Sigma) (25 injections), the GABA antagonist picrotoxin (Sigma) was applied in 10 of those cases. Bumetanide (Sigma) was used to block the chloride NKCC cotransporters (21 injections). Furosemide was applied to block the chloride cotransporter KCC2 (8 injections). Stem solutions of the chemicals were prepared and stored at −20°C until use. Shortly before use, stem solutions were diluted with 0.9% saline (NaCl). Concentrations of stem solution, solvents, working solutions, and estimated final concentrations are summarized in Table 1.

**Table 1:** Table 1 summarizes the solvents used for the stem solutions, the concentration of the stem solutions, the concentration of the working solutions, the volume injected, the concentration in the vitreous body based on an estimated volume of 50 µl [50] up to 100 µl (own measurements in older animals (>400 g), and the number of tests performed.

substance	solvent	stem solution	working solution	volume	concentration in vitreous	tests (effective)
bumetanide	ethanol	100mM	300µM	10µl	30–60µM	21 (17)
furosemide	acetone	25mM	250µM	10µl	25–50µM	8 (7)
APB	0.9% NaCl	55mM	0.4–10mM	5–10µl	40–200µM	25 (22)
PTX	0.1M PBS	2.5mM	1mM	5–10µl	100–200µM	10 (10)

Numbers in parentheses give the number of tests where drug administration resulted in changes of the neuronal activity. APB: 2-amino-4-phosphonobutyrat; PTX: picrotoxin; PBS: phosphate buffered saline.

To verify that changes in neuronal response properties were not artefacts of the injection technique we performed a control experiment using 0.9% NaCl. There was no alteration in response properties after 3 10 µl injections indicating that the changes described below were indeed due to the pharmacological substances injected.

### Cortex inactivation

As a further control, in 3 animals visual cortex was inactivated to determine the cortical influence on the ON- and the OFF response. For this purpose, a plastic chamber was fixed to the skull and visual cortex was cooled with a mixture of glycerol and the blood substitute FC-43 emulsion (The Green Cross Co.) as 3∶1. Fluid temperature in the chamber was initially −5°C to −10°C. After cortical activity recorded with a microelectrode was abolished, direction selectivity, ON/OFF response, and velocity tuning in the NOT-DTN was tested in 9 units. After these measurements, the cortex was warmed with 41°C NaCl and additional measurements of the NOT-DTN cells were conducted. Effectiveness and reversibility of this cooling method was verified by recording cortical activity in the cooled region.

### Data analysis

Peri-stimulus-time histograms were analyzed using Matlab® 7.2.0 (The MathWorks, Inc). The direction selectivity of the neurons was characterized by a direction selectivity index (DSI) calculated as follows: DSI = (PD-NPD)/PD, with PD representing the activity during stimulation in the preferred direction, and NPD the activity during stimulation in the opposite non-preferred direction ( = nulldirection). An index around 1 indicates high direction selectivity, an index around 0 no direction selectivity. For statistical analysis we applied an unpaired t-test for normally distributed values, a Wilcoxon signed rank test for paired comparison, and a Mann-Whitney rank sum test for all other data sets. Figures were prepared using SigmaPlot vs. 8.0.

## Results

**Figure 2:**
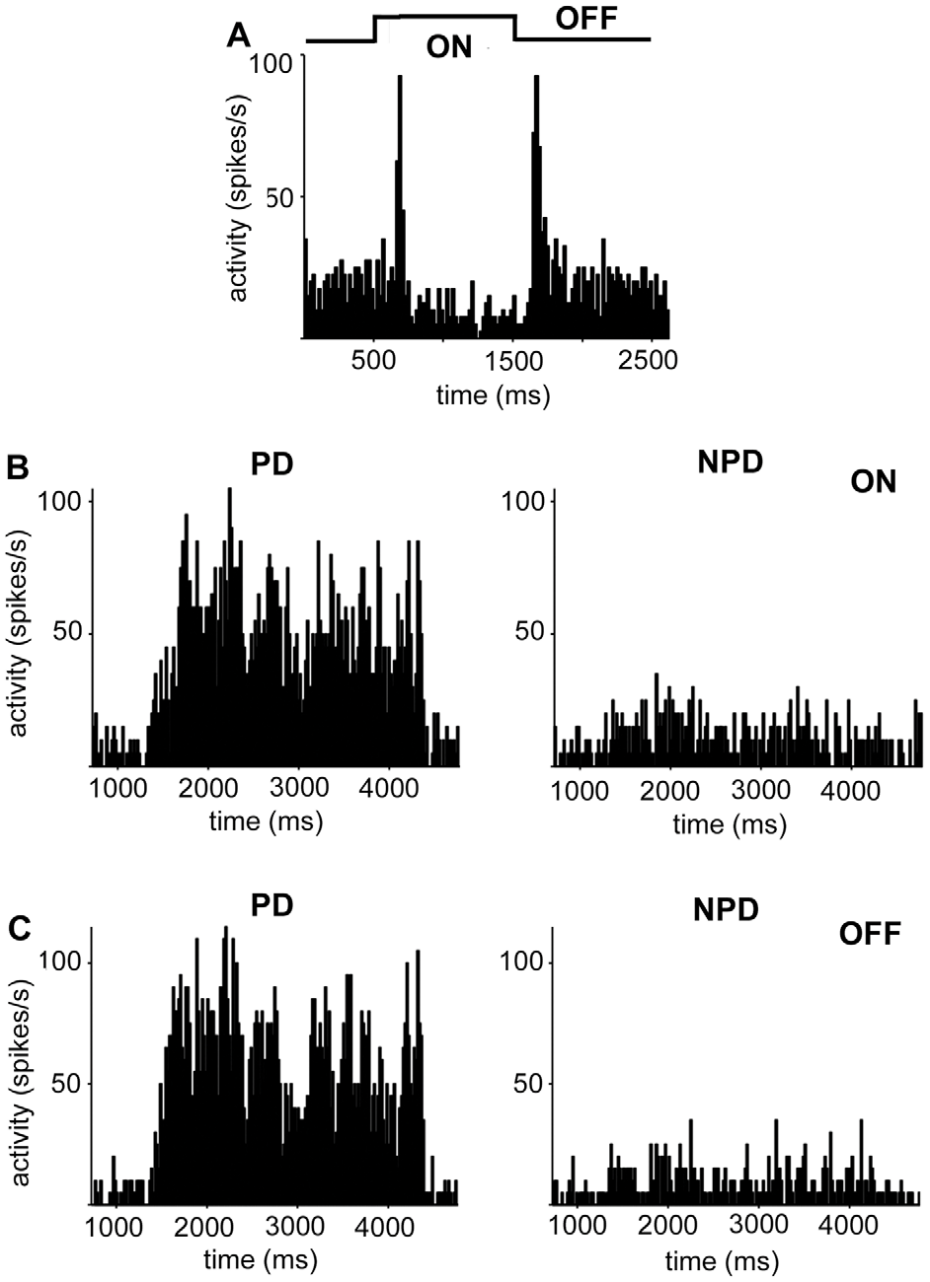
NOT-DTN neurons respond to flashed as well as moving ON- and OFF visual stimuli. A: Response to flash stimulation, light onset at 500 ms, light offset at 1500 ms. This neuron exhibits clear phasic responses to both ON and OFF stimulation, the tonic response is low during ON stimulation. Spontaneous activity is represented by the first 500 ms of the PSTH. Ordinate: activity in spikes per second (spikes/s), abscissa: time in milliseconds (ms). B: direction selective response to a moving light edge (ON). Movement onset at 1000 ms. PD: preferred direction, NPD: non-preferred direction (opposite to PD). C: direction selective response to a moving dark edge (OFF).

Altogether 94 recordings of single and multiple retinal slip neurons in the NOT-DTN were performed. However, only those 64 recordings containing neurons with a DS index higher than 0.3, and with peak discharge rates higher than 10 spikes/s in the preferred direction were included in the analysis. Single cells and multi-units were analyzed separately. The data base of the present study is summarized in Table 1. Figure 2 demonstrates exemplary NOT-DTN neurons' responses to a stationary ON and OFF flash stimulus (Fig. 2A), to a moving ON stimulus (Fig. 2B), and to a moving OFF stimulus (Fig. 2C). It is evident that retinal slip cells in the NOT-DTN have a pronounced direction selective response to moving ON as well as OFF edges. These responses are not due to or changed qualitatively by the different temporal gradients in mean illumination with the ON (increasing mean illumination) or OFF stimulus (decreasing mean illumination). Movements perpendicular to the preferred – non-preferred axis which changed the intensity of the mean illumination in the same way as motion in the preferred direction changed the ongoing discharge rate of retinal slip cells only insignificantly or not at all. Also stimulation with different mean illumination of the computer screen (sine wave grating versus ON and OFF edges) or adding background illumination (room light: 60 cd/m^2^) had no qualitative effect on the directional tuning of the ON- and OFF responses to moving stimuli. To elucidate the origin of this OFF response we performed the following experiments.

### Cortical inactivation

In all eutherian mammals tested so far, the NOT-DTN receives input from visual cortex. Visual cortex relays binocularity and response to high stimulus velocities to the NOT-DTN and the optokinetic system [for review 10]. Furthermore, in cat blockade of the retinal ON-system with APB eliminates hOKR only if visual cortex is eliminated [13]. A cortical input has also been demonstrated in the rat [1], its functional significance, however, remains unclear because cortical lesions do not affect hOKR in the rat [38].

**Figure 3:**
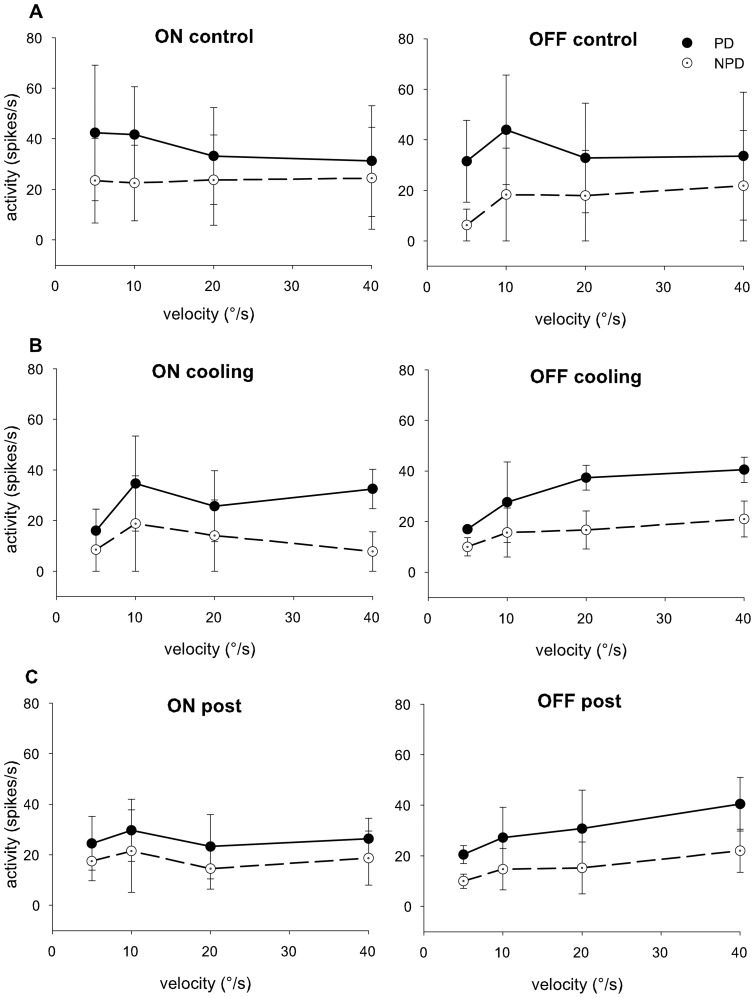
Effects of cortical inactivation on ON and OFF reponses. Direction selective responses to a light edge (left panels) or a dark edge (right panels) moving at velocities from 5–40°/s in preferred (filled circles, solid black line) and non preferred (open circles and broken line) directions. Mean and standard deviation from 5 units are plotted, control measurements in A, measurements during cooling in B, and after rewarming in C. ordinate: neuronal activity in spikes/second; abscissa: stimulus velocity in degrees/second. There was no significant alteration of response strength and direction selectivity by cortical cooling at any of the velocities tested.

In order to test if and to what extent the OFF response of retinal slip cells in the NOT-DTN in the rat depends on cortical input we reversibly inactivated this projection by cooling. We compared the neuronal responses of 9 retinal slip units before and after cortical inactivation. There was no significant alteration of the response strength and of the DSI in the ON or OFF responses over the velocity range tested by the cortical inactivation (Fig. 3). This clearly indicates that retinal or subcortical inputs are sufficient to create the direction selective ON as well as the OFF response of retinal slip cells in the NOT-DTN of rats.

### Blocking the retinal ON pathway with APB

**Figure 4:**
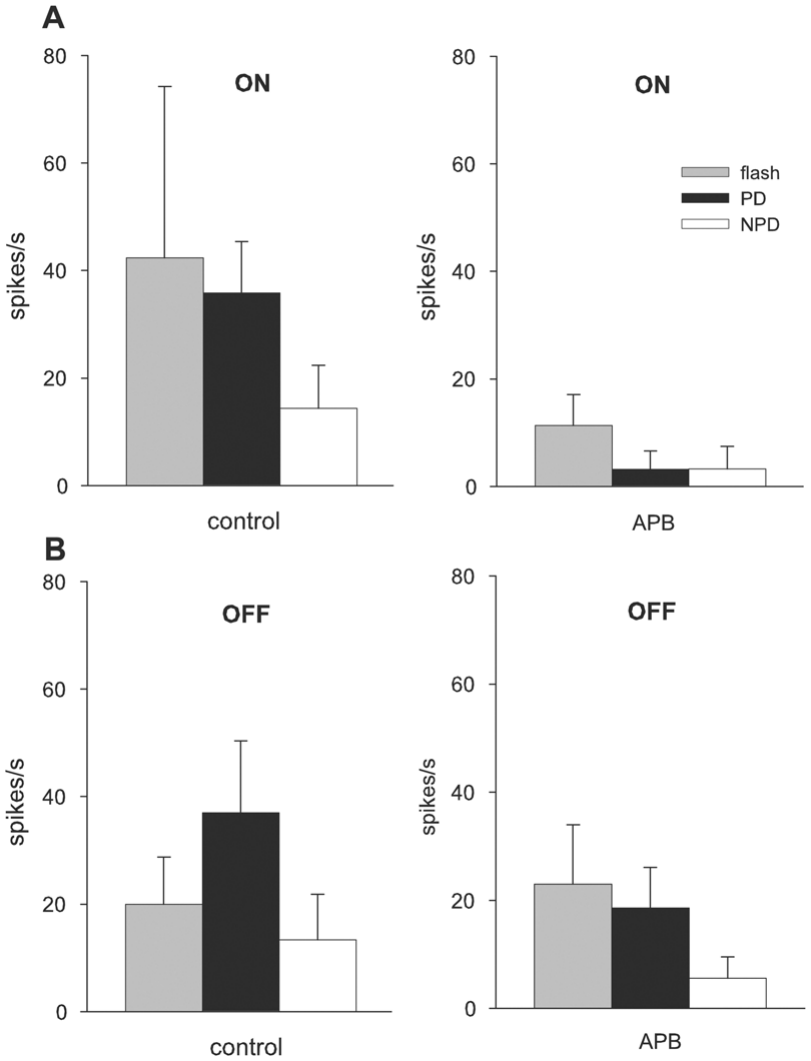
Effects of 2-amino-4-phosphonobutyrat (40–80 µM APB) on neuronal activity of single units. Responses of 3 units (mean and standard deviation of 10 measurements) to flashed and moving ON (A) and OFF (B) visual stimuli before (control) and after intravitreous drug injection (APB; intravitreous concentration 40–80 µM). Ordinate: neuronal activity in spikes per second, abscissa: experimental conditions. APB blocks responses to all ON stimuli but spares the OFF flash response and the responses to the moving dark edge, albeit moderately decreased. Grey boxes: flash responses; black boxes: responses to moving edges in preferred direction; white boxes: responses in non-preferred direction.

To test whether the OFF response in the NOT-DTN resides on the retinal ON pathway 2-amino-4-phosphonobutyrate (APB) was injected intraocularly. As expected, with an intravitreous concentration of 40–80 µM APB, all 3 NOT-DTN neurons tested showed a significant reduction of the ON flash response (Fig 4A) but no change or a slight not significant increase of the OFF flash response. Responses driven by the moving ON stimulus were dramatically reduced (p<0.01; t-test, n =  measurements) during PD and NPD stimulation resulting in a complete loss of direction selectivity (Fig. 4A). With this intravitreous concentration, a direction selective OFF response was retained despite a decrease of mainly the responses in PD (Fig. 4B).

**Figure 5:**
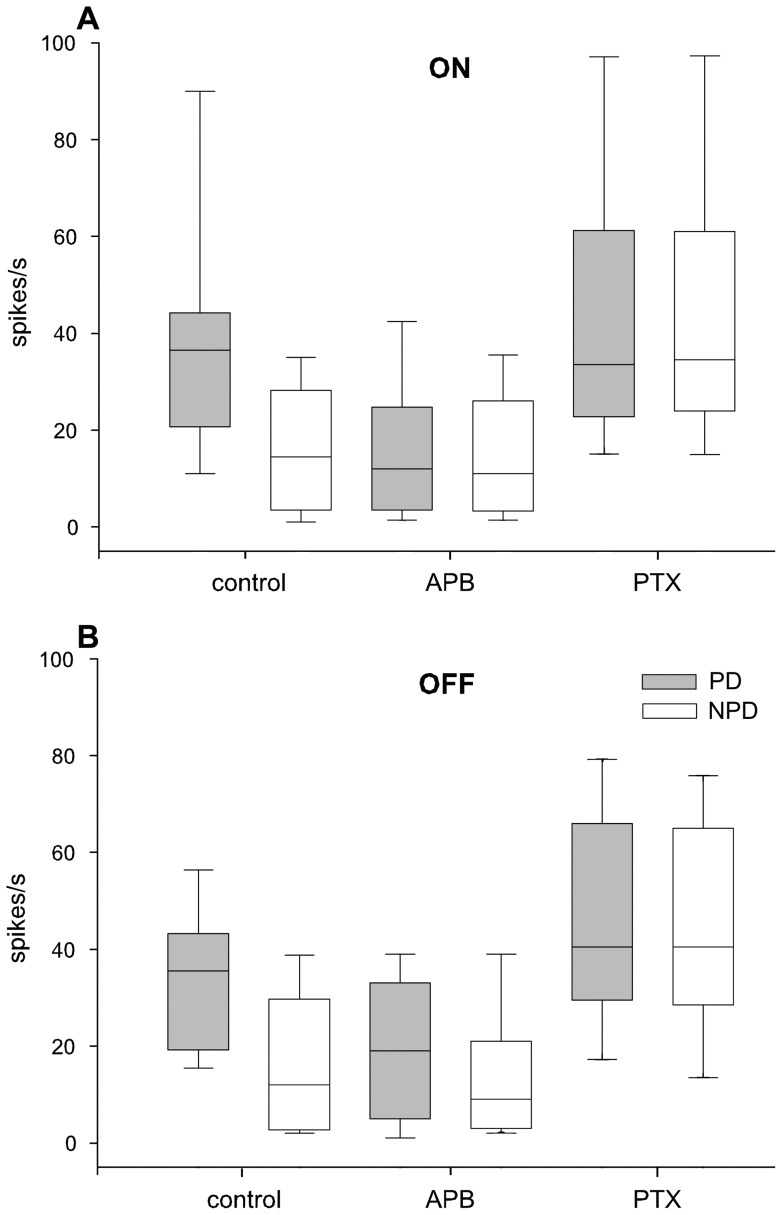
Effects of 2-amino-4-phosphonobutyrat (APB) and picrotoxin (PTX) on neuronal activity in all units tested. Neuronal responses in all 25 single- and multi-unit recordings during ON A) and OFF (B) stimulation in preferred (PD, grey boxes) and non-preferred (NPD, white boxes) direction before (control; left 2 boxes), after intravitreous application of the drug APB (middle 2 boxes) and after subsequent intravitreous application of PTX (right 2 boxes). Horizontal lines indicate the median, boxes the 25–75%, and whiskers the 10–90% percentiles of non-parametric statistical comparison. Ordinate: neuronal activity in spikes per second, abscissa: experimental conditions. At approximately 100–200 µM intravitreal concentration, APB completely blocks the response driven by the moving ON stimulus (p<0.005) but also decreases the response to the moving OFF stimulus (p<0.05). The activity in the non-preferred direction is unaltered as is spontaneous activity. PTX increases the responses in PD and in NPD to about the same values during ON- as well as during OFF stimulation.

To make sure that we blocked the retinal ON system completely we increased the APB dosage in the following experiments to 100–200 µM. Surprisingly, now also the OFF response was reduced in 11 out of 14 multi units and in all 8 single units tested during PD stimulation but remained largely unaffected during NPD stimulation (Fig. 5). However, the reduction was significantly (p<0.001) stronger in the ON response (down to 25%) than in the OFF response (down to 49% of the control measurements). In a pairwise comparison (Wilcoxon signed-rank test) including data from all 11 single- and 14 multi-unit recordings APB had a significant effect on the responses in the preferred direction for the ON and OFF stimulation (p<0.0001), on the response in the non-preferred direction for the ON (p<0.005) but not for the non-preferred OFF stimulation (p>0.1). When responses in preferred and non-preferred direction were compared after intravitreal APB injection direction selectivity was lost during ON (p>0.2) but not during OFF-stimulation (p<0.005). During flash stimulation both ON and OFF responses were eliminated. APB had no effect on the spontaneous activity of our multi-units (control 28 spikes/s, APB 25 spikes/s, p-0.748) but led to an increase in spontaneous discharge in our sample of single units (control 2 spikes/s, APB 11 spikes/s, p<0.001).

**Figure 6:**
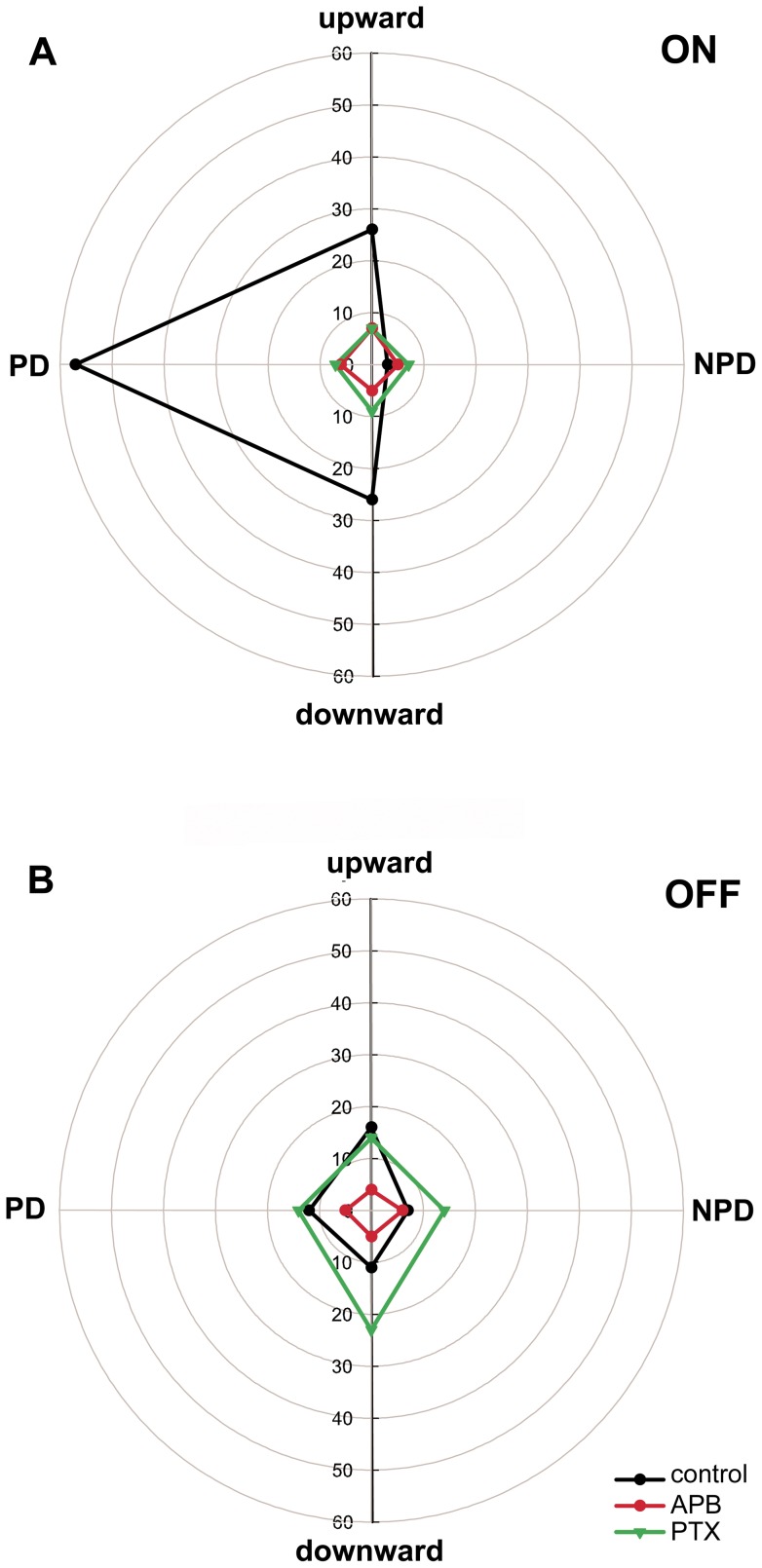
Effects of 2-amino-4-phosphonobutyrat (APB) and picrotoxin (PTX) on the directional tuning of the light edge and dark edge responses in an example NOT-DTN unit. Polar plot of the responses during stimulation with ON (A) and OFF (B) edges moving in the four cardinal directions prior to drug application (black curves), after intravitreous injection of APB (red curves), and after additional intravitreous injection of PTX (green curves). Distance from the center of the polar plots indicates neuronal activity in spikes per second given by the numbers on the circles. PD: preferred horizontal direction, NPD: non-preferred horizontal direction. Note that after APB followed by a PTX injection an increased but direction unselective response to the moving dark edge (OFF) is uncovered.

In retinal ON center direction selective ganglion cells picrotoxin (PTX) unmasks OFF responses with a PD in the opposite direction of the ON response after the ON response was blocked with APB in the rabbit [37]. To determine if the OFF response of NOT-DTN retinal slip cells in the rat could be mediated by the ON retinal ganglion cells we blocked the GABA_A_,GABA_C_ and glycine receptors with PTX after blocking the ON channel with APB in 10 cases. The directional tuning of a unit with weak OFF response showed a preference for leftward stimulus movement in both the ON (Fig. 6A) and, albeit weaker, in the OFF response (Fig. 6B) prior to the injections (black curves). After 200 µM APB injection, the ON and OFF responses were blocked and direction selectivity was lost (red curves). After additional PTX injection the OFF response exceeded the values of the control measurements prior to the APB injection with a slight preference for downward stimulation (Fig. 6B, green curves). PTX had little and unspecific effects on the ON responses relative to the APB effect (Fig. 6A). In fact, in most cases PTX application decreased the PD response and increased the NPD response compared to the controls thus not restoring direction selectivity during ON stimulation. The same was found during OFF stimulation. PTX increased the responses in PD and NPD. Thus, PTX applied to the retina after APB unmasked prominent but direction unselective ON – and OFF responses in all 10 NOT-DTN retinal slip cells tested (Fig. 5; right 2 boxes in A and B). The direction specific effect of PTX after APB in the OFF response similar to that described for ON center direction selective ganglion cells in the retina [37] could not be exposed in the NOT-DTN.

### Manipulation of the retinal chloride homeostasis: bumetanide

To determine whether direction selectivity of the ON and the OFF response in the NOT-DTN depends on the direction selectivity of starburst amacrine dendrites in the retina [26], [29] or is created *de novo* in the NOT-DTN we manipulated the retinal chloride equilibrium. Intraocular injection of bumetanide with intravitreal concentrations of approximately 60 µM should reduce the action of mainly the chloride inward cotransporter NKCC2 which should lead to a loss of direction selectivity of the starburst amacrine dendrites [30].

**Figure 7:**
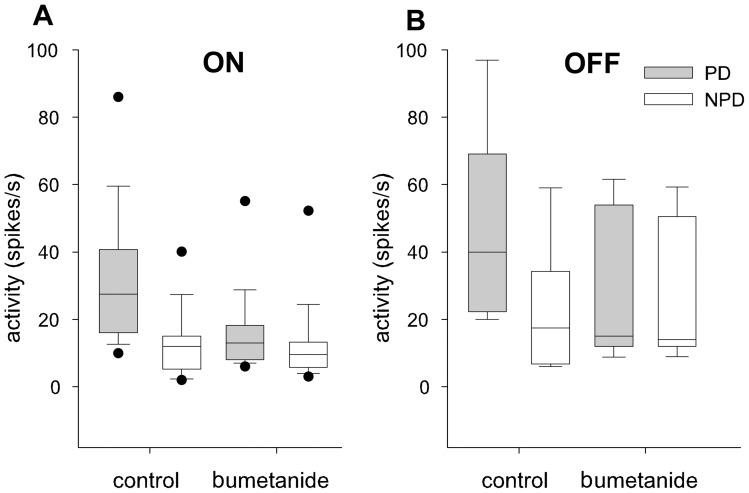
Effects of intravitreal bumetanide injections on the responses of NOT-DTN neurons to moving light (ON) and dark (OFF) edges. Comparison of the activity during ON (A) and OFF (B) moving edge stimulation in preferred (PD, grey bars) and non-preferred (NPD, white bars) directions prior to (control) and after intravitreous injection of the drug (bumetanide). Horizontal lines indicate the median, boxes the 25–75%, whiskers the 10–90%, and black dots the 5–95 percentiles of the non-parametric statistical comparison. Bumetanide reduces responses in preferred direction and enhances responses in the non preferred direction, especially in the responses to the OFF stimulus.

**Figure 8:**
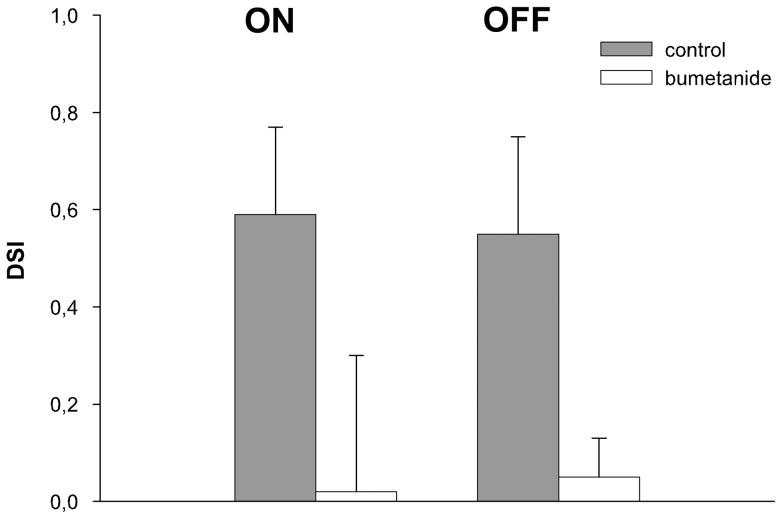
Effects of bumetanide on direction selectivity. Comparison of the direction selectivity index DSI (ordinate) during ON and OFF stimulation before (grey columns) and after (white columns) intravitreous application of bumetanide averaged (mean and standard deviation) over all units recorded. Bumetanide abolishes direction selectivity in the retina and subsequently in NOT-DTN retinal slip cells.

We analyzed the effects on stimulus driven ON and OFF activity, direction selectivity, and spontaneous activity averaged over several measurements in the NOT-DTN after the injections. Of 21 tests with bumetanide injection, 17 demonstrated altered response properties. A pairwise comparison of single and multi-unit responses proved that bumetanide caused a significant reduction of the stimulus driven activity in PD during ON (p<0.0001) and OFF stimulation (p<0.05) (Fig. 7). Neuronal activity during stimulation in NPD was elevated in some neurons but not significantly at the population level (p>0.8) (Fig. 7). The reduced activity in PD and the elevated activity in NPD resulted in a highly significant drop in the DSI both for the ON (p = 0.002) and the OFF stimulus (p≤0.005) (Fig. 8). Bumetanide had no significant effect on the spontaneous discharge rate (multi-unit: control 35 spikes/s, bumetanide 50 spikes/s, p = 0.44, single unit: control 12 spikes/s, bumetanide 13 spikes/s, p = 0.517).

### Manipulation of the retinal chloride homeostasis: furosemide

**Figure 9:**
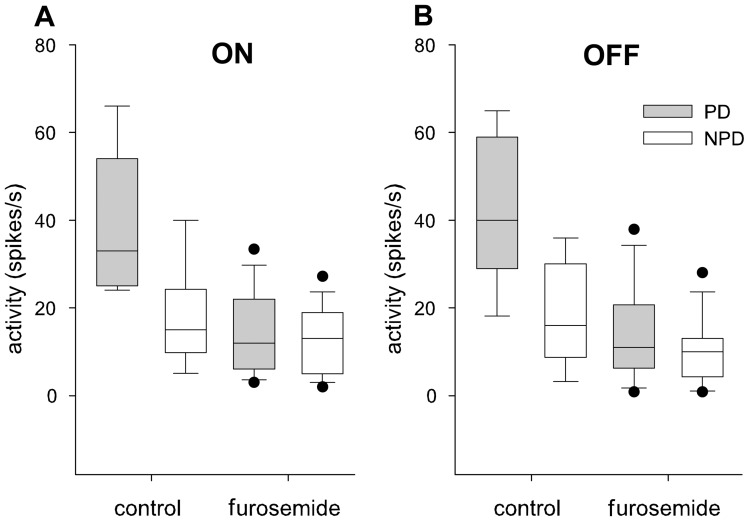
Effects of intravitreal furosemide injections on the responses of NOT-DTN neurons to moving light (ON) and dark (OFF) edges. Comparison of the responses during ON (A) and OFF (B) moving edge stimulation in preferred (PD, grey bars) and non-preferred (NPD, white bars) directions prior to (control) and after intravitreous injection of the drug furosemide. Horizontal lines indicate the median, boxes the 25–75%, whiskers the 10–90%, and black dots the 5–95 percentiles of the non-parametric statistical comparison. Furosemide reduces responses in preferred and in non preferred direction.

**Figure 10:**
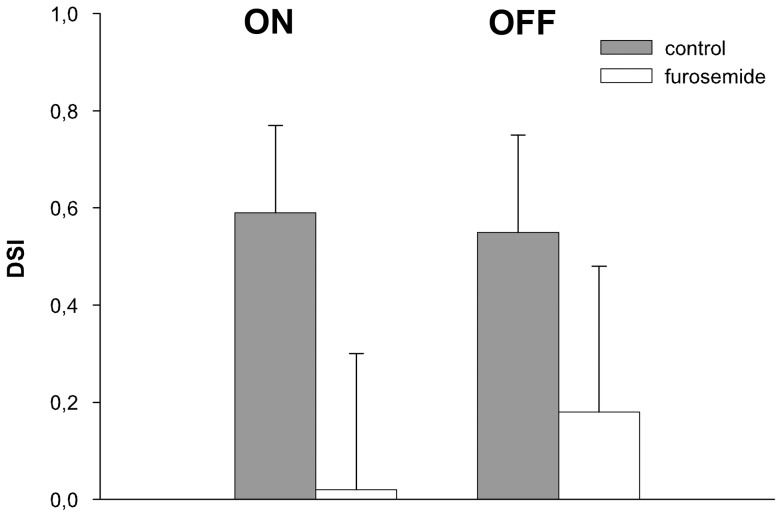
Effects of furosemide on direction selectivity. Comparison of the direction selectivity index DSI (ordinate) during ON and OFF stimulation before (grey columns) and after (white columns) intravitreous application of furosemide. averaged (mean and standard deviation) over all units recorded. Furosemide abolishes direction selectivity in the retina and subsequently in the ON and to a lesser degree in the OFF responses in NOT-DTN retinal slip cells.

The retinal chloride equilibrium was further manipulated by decreasing the action of the chloride outward cotransporter KCC2 with furosemide. In 7 out of 8 units, intravitreal injection of furosemide (concentration in the vitreous 25–50 µM) resulted in a clear reduction of activity in PD during ON and OFF stimulation (Fig. 9). In a pairwise comparison of single and multi-unit responses furosemide caused a significant reduction of the stimulus driven activity during stimulation in PD during ON (p<0.01) and OFF stimulation (p<0.01). No significant changes occurred in NPD (p>0.83 for the ON, p = 0.57 for the OFF stimulus) (Fig. 9B). These effects resulted in a significant reduction of the DSI during ON (p = 0.012) and OFF stimulation (p = 0.017) (Fig. 10). In no case did we observe full recovery of the neuronal response over the time period measured. The reduced stimulus driven activity was accompanied by a highly significant decrease in the spontaneous activity (multi-unit: median control 26 spikes/s, furosemide 10 spikes/s, p<0.001; single cell: control 11 spikes/s, furosemide 1 spike/s, p<0.001).

Thus, manipulation of the retinal chloride equilibrium that presumably reduces direction selectivity of the output of starburst amacrine cells results in a reduction or loss of direction selectivity of the ON as well as the OFF response in NOT-DTN retinal slip cells.

## Discussion

In the present study we could manipulate the response properties of retinal slip cells *in vivo* by intravitreal injections of various substances selectively affecting different substrates of retinal information processing. By employing a visual stimulus consisting of large black and white edges we were able to dissociate an ON as well as an OFF response in NOT-DTN retinal slip cells and analyze them individually. Control experiments inactivating visual cortex by cooling revealed that the OFF response was not mediated by the visual cortex. The relatively unchanged responses over a velocity range of 5–40°/s are in agreement with the unchanged OKN after decortication in the rat [38].

There is a wealth of data showing that ON center direction selective ganglion cells project to nuclei of the accessory optic system. Blocking the retinal ON pathway leads to a loss of OKR in frog [39], turtle [40], chicken [41], and rabbit [13]. In addition, at APB concentrations of 50–200 µM direction selective responses to moving stimuli are blocked in the turtle basal optic nucleus [40], and direction selectivity in the rat NOT-DTN is cancelled (this study). The unexpected part of our results is that not only the ON but also the OFF response was abolished after APB injection. The effect on the OFF response was dosage dependent: the response was reduced at 100–200 µM but less affected and still direction selective at 40–80 µM APB. Similarly, in retinal ON-OFF direction selective ganglion cells also only the ON response was affected by 100 µM APB, the OFF response remained direction selective [42]. Antidromic stimulation identified ON as well as ON-OFF direction selective ganglion cells to project to the cat's NOT-DTN [12]. Oyster et al. [11] and Collewijn [43] argued that the velocity range effective to drive OKR and direction selective responses in the NOT of rabbits require input from both ON direction selective ganglion cells for slow, and ON-OFF direction selective ganglion cells for fast retinal image motion. There is, however, no evidence that the retinal OFF system alone can drive the optokinetic reflex if there is no additional input from visual cortex [13], [39], [44]. Why then abolish intravitreal injections of APB the horizontal OKR in cats after decortication and in rabbits if the NOT-DTN receives retinal input from an APB resistant direction selective OFF system? Our data in the rat clearly show that direction selectivity of the OFF responses in the NOT-DTN is strongly diminished by intraretinal APB. Thus, there must be a mutual facilitatory influence between the ON- and the OFF system either in the retina or in the NOT-DTN to drive OKR. This suggestion is in line with the conclusions reached by Ibbotson and Clifford [45] studying the interactions of ON- and OFF-responses in NOT neurons of the wallaby. It would have been very informative to investigate the effects of blocking the OFF input on responses in the NOT-DTN and on OKR. Unfortunately, the glutamate antagonist cis-2,3-piperidinedicarboxylic acid (PDA) was not specific enough in our hands to eliminate the OFF response while leaving enough of the ON response intact [46]. In 5 injections PDA (intravitreal concentration 8–10 µM) always reduced OFF as well as ON responses and removed direction selectivity in the NOT-DTN.

It is well established that GABAergic mechanisms are crucial for the generation of direction selectivity [for review e.g. 27,47]. Blocking GABA receptors not only leads to a loss of direction selectivity in ganglion cells [42], [48], [49] but in addition unmasks an OFF response in ON direction selective ganglion cells [37] which is selective for visual motion in the direction opposite to that of the ON response. It has been proposed that this unmasked OFF response is mediated from polyaxonal amacrine cells via gap junctions to the ON direction selective ganglion cells [37]. To further examine the origin of the OFF response in retinal slip cells we blocked GABA receptors with picrotoxin after having inactivated the retinal ON channel with APB. This treatment greatly enhanced the OFF response in the NOT-DTN but also rendered it direction unselective.

It is difficult to know the actual concentration of pharmacological agents at synaptic sites in the retina following intraocular injection. Our estimate of the concentrations was based on 50 µl of vitreous [50] in young rat's eyes but in older animals the volume could be up to 100 µl (own post-mortem estimate). Still our concentration of picrotoxin of 100 to 200 µM is rather high and blocks GABA_A_, GABA_C_ and glycine receptors. Further experiments using concentrations as low as 20 µM with a selective antagonism to GABA_A_ and GABA_C_ receptors are needed to study the unmasking of the OFF response by picrotoxin. But another caveat remains. Since there was an OFF response with the same directional preference as the ON response in the NOT-DTN even before picrotoxin was applied to the retina the unmasked OFF response by picrotoxin might indeed have had the opposite directional preference and the addition of the two responses created an unselective response. Alternatively, our contradictory result could most easily be explained by the fact that there exist more than one type of ON direction selective ganglion cells as was recently described in the rabbit [51], [52], and if the one analyzed by Ackert et al. [37] does not project to the NOT.

Taken together our results support the hypothesis that the OFF response of retinal slip cells does not originate as a rebound from the retinal ON system but may be derived from subthreshold OFF input from amacrine cells to the direction selective ON ganglion cells projecting to the NOT-DTN. These results seem to further support the notion that retinal ganglion cells may not exclusively receive input from either ON or OFF bipolars and amacrine cells but that inputs from both systems may be combined even if one of them dominates [37], [45], [53], [54], [55], [56], [57]. Moreover, the NOT-DTN may receive an additional as yet undetected input from OFF direction selective ganglion cells and from ON/OFF ganglion cells as recently described for the mouse [21]. Alternatively, local mechanisms in the NOT-DTN enhancing normally silent neuronal connections in case of a decreased ON input may cause our increased OFF response [58].

In order to investigate the relative role of the direct retinal input to the NOT-DTN for direction selectivity of its retinal slip cells we attempted to eliminate the direction selective release of GABA from dendrites of starburst amacrine cells by blocking the cation-chloride cotransporters NKCC2 and KCC2 responsible for the differential intracellular chloride concentration along the starburst amacrine dendrites. NKCC1 can be selectively blocked by 1–10 µM bumetanide which has a more than 100-fold higher affinity for NKCC1 than for KCC2. KCC2 is blocked by furosemide. It is often used at 100 µM or higher which still does not provide a full block, and an obvious confounding factor is the simultaneous effect on NKCC with an equal potency [59], [60].

We attempted to apply similar intravitreal concentrations as applied in *in vitro* preparations of the retina by Gavrikov et al. [30], [31]. They showed that bumetanide hyperpolarized starburst amacrine cells by blocking selectively NKCC2 and eliminated their direction selective response to motion. The direction selective ganglion cells were then excited by stimulus motion in all directions. In contrast, furosemide depolarized starburst amacrine cells by blocking KCC2, eliminated their hyperpolarizing response to centripetal motion, and reduced but did not abolish their depolarizing response to centrifugal motion. The direction selective ganglion cells would then respond to a lesser extent to stimulus motion in all directions. The selective inhibitory effect of furosemide on starburst KCC2 compared with NKCC2 was shown by the consistently opposite effects of furosemide and bumetanide. Gavrikov et al. [31] argue that starburst amacrine cells express a furosemide-insensitive NKCC2 subtype.

Indeed, in our experiments after injection of furosemide retinal slip activity during stimulation in the preferred direction as well as the direction selectivity index of both the ON- as well as the OFF response were significantly reduced meeting the expectation from the furosemide effect on starburst amacrine cells. After injection of bumetanide the results do not correspond so well to the retinal effects. Activity during stimulation in the non-preferred direction (especially the OFF response) as well as spontaneous activity were somewhat enhanced but activity in the preferred direction was reduced. A reduction of activity during PD stimulation was not described for bumetanide but only for furosemide by Gavrikov et al. [30], [31]. In our *in vivo* approach additional influences from cells other than starburst amacrines have to be taken into account. Likely candidates are bipolar cells that contain chloride cotransporters in their dendrites and axon terminals [61]. Our results clearly indicate that disturbing the retinal chloride homoeostasis and thus presumably eliminating retinal direction selectivity is sufficient to abolish direction selectivity in the NOT-DTN thereby strengthening the notion that the direction selectivity in the NOT-DTN is mainly caused by the retinal input. These results also suggest that the direction selective OFF response in the NOT-DTN does not come from extraretinal sources like the visual cortex but from the retina because it is also dependent on intraretinal chloride transporters.
